# Comparison Between Eleven-Bar Cushion and Pillow for Contrast Media Spread in Caudal Block

**DOI:** 10.3390/jcm14238524

**Published:** 2025-12-01

**Authors:** Jaeho Cho, Sang Jun Park, Jae Chul Koh, Na Eun Kim, Won Sok Chang, Jae Hyung Kim, Keuntak Yuk, Mazen Zein, Jong Bum Choi, Yi Hwa Choi

**Affiliations:** 1 Department of Anesthesiology and Pain Medicine, Ajou University School of Medicine, Suwon 16499, Republic of Korea; jaehotv@gmail.com; 2 Department of Anesthesiology and Pain Medicine, Yonsei University School of Medicine, Severance Hospital, Seoul 03711, Republic of Korea; 3 Department of Anesthesiology and Pain Medicine, Korea University School of Medicine, Anam Hospital, Seoul 02841, Republic of Korea; jaykoh@korea.ac.kr; 4 Department of Anesthesiology and Pain Medicine, Inha University School of Medicine, Incheon 22332, Republic of Korea; friskygirl@naver.com; 5 Department of Anesthesiology and Pain Medicine, Chungdam Wooridul Spine Hospital, Seoul 06068, Republic of Korea; knowspine@naver.com; 6 Department of Anesthesiology and Pain Medicine, Hallym University School of Medicine, Dongtan Sacred Heart Hospital, Hwaseong 17153, Republic of Korea; jaehkim11@gmail.com (J.H.K.); keuntak@hallym.or.kr (K.Y.); 7 Department of Neurosurgery, Division of Spine Medicine, Duke University School of Medicine, Durham, NC 27708, USA; mazen.zein@duke.edu; 8 Department of Anesthesiology and Pain Medicine, Hallym University School of Medicine, Anyang 14068, Republic of Korea

**Keywords:** caudal epidural steroid injection, fluoroscopy, injectate spread, lumbar spine, epidural block, positioning, eleven-bar cushion

## Abstract

**Background/Objectives:** A caudal epidural steroid injection (CESI) is a widely used technique for managing low back and lower extremity pain due to its relative ease and safety. However, cephalic spread of the injectate may be limited by the long distance from the sacral hiatus and by increased intra-abdominal pressure caused using conventional abdominal pillows during prone positioning. This study aimed to investigate whether an eleven-bar cushion could facilitate higher cephalic spread of contrast medium during CESI compared to a conventional pillow. **Methods:** This retrospective study was approved by the Institutional Review Board (IRB number: AJOUIRB-DB-2025-103). Data from 76 patients, who underwent CESI between January 2023 and March 2024, were analyzed. Patients were divided into two groups the eleven-bar group (n = 38) using a pelvic eleven-bar cushion and the pillow group (n = 38) using a conventional pillow. Fluoroscopic images were reviewed to identify the highest vertebral level reached by the injectate and the number of nerve roots visualized. Visual analogue scale (VAS) scores before and one month after the procedure were also assessed. Statistical analyses included Mann–Whitney U tests, linear regression, and Poisson regression. **Results:** Baseline demographic characteristics were similar between groups. The cephalic spread of contrast medium was significantly higher in the eleven-bar group compared with the pillow group (median level L3/4 vs. L4/5, *p* = 0.0002). No significant differences were observed in the number of nerve roots reached or in the VAS score improvement between groups. **Conclusions:** The eleven-bar cushion facilitated greater cephalic spread of contrast medium during CESI compared with a conventional pillow. Although this technique did not affect nerve root distribution or pain reduction outcomes, it may represent a useful positioning strategy to enhance drug delivery to higher lumbar levels during caudal epidural injections.

## 1. Introduction

A CESI has been shown to be an effective treatment approach for low back and/or lower extremity pain [1,2,3,4]. It is considered a relatively easy technique in the interventional pain management field and is also known to present a lower risk of accidental dural puncture than other epidural techniques [5]. The relatively low cost for a caudal epidural compared with that of transforaminal epidural injections is another important consideration.

**Figure 1:**
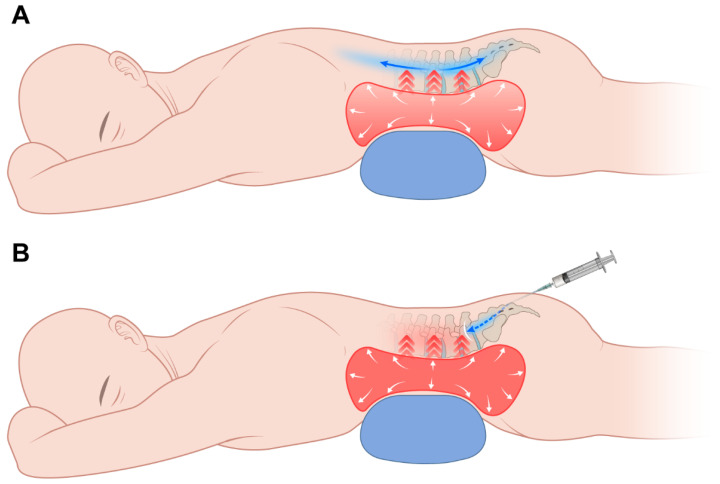
Schematic illustration showing the placement of a pillow under the abdomen and its effect on caudal block. (**A**) Injection before increasing intra-abdominal pressure by pillow. Injectate may spread widely. (**B**) Injection after increasing intra-abdominal pressure by pillow. Impeded cephalic spread of injectate.

However, despite its apparent safety, caudal epidural injection has a significant limitation. Its injectate often fails to reach the desired lumbar level due to the long distance from the sacral hiatus and the loss of volume caused by leakage into the sacral foramina [6,7]. Moreover, lumbar pathology can result in resistance to injectate spread in the epidural space [8]. Epidural fibrosis and adhesions are considered to interrupt nerve root blood supply, which can prevent contact of the injectate with the affected nerve root [9]. In addition, after injection of spinal or epidural block, increased abdominal pressure after spinal or epidural injection may promote broad injectate distribution. However, placing a conventional pillow under the abdomen increases intra-abdominal pressure, which can impede cephalad injectate spread during caudal injection (Figure 1).

Several studies have attempted to achieve a higher spread of the injectate along the lumbar spine. However, there is no study about potential strategies to improve cephalad injectate distribution by using a supporting cushion in the prone position.

**Figure 2:**
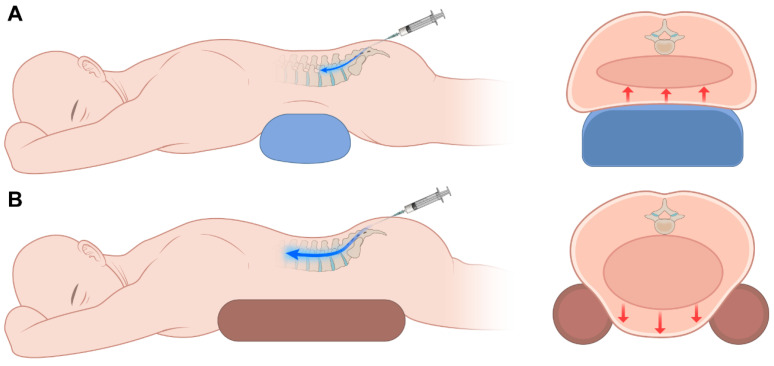
Schematic illustration showing that the use of an eleven-bar cushion reduces abdominal pressure, thereby facilitating greater cephalic spread of contrast medium using (**A**) a pillow and (**B**) an eleven-bar cushion.

This study aimed to determine whether reducing lumbar lordosis and lowering abdominal/epidural pressure using the eleven-bar cushion would allow the injectate to reach higher vertebral levels on epidurography compared with a conventional pillow (Figure 2).

## 2. Materials and Methods

This retrospective study was performed after obtaining Institutional Review Board approval. from the Institutional Review Board at our hospital (IRB number: AJOUIRB-DB-2025-103). Data were collected from January 2023 to March 2024 in an outpatient pain clinic. Exclusion criteria included incomplete fluoroscopic images, combined epidural approach, non-standard injectate volume. The evaluation included demographic data of age, gender, weight, height, symptoms (low back pain or radiating pain or both), and history of lumbar spine surgery.

**Figure 3:**
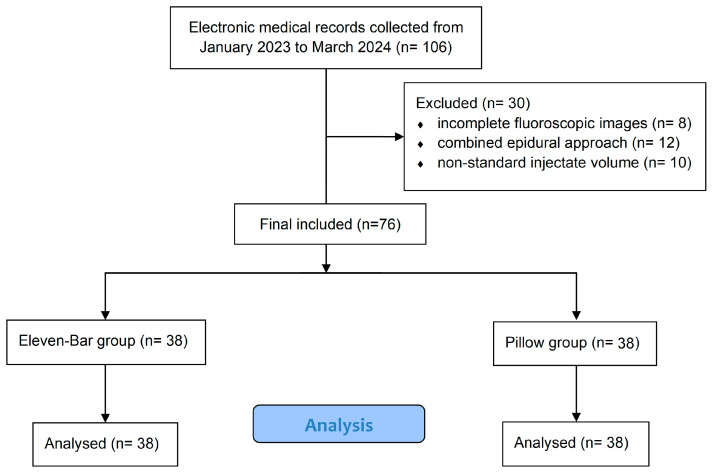
STROBE flow diagram of patient selection.

**Figure 4:**
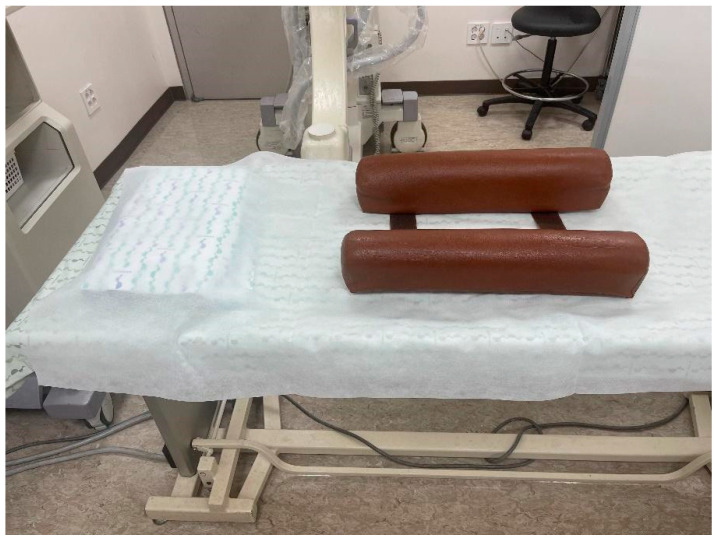
This is a picture of eleven-bar cushion setting on the operating room bed for the caudal block.

Seventy-six patients were enrolled in the study. The patient selection process is summarized in the STROBE flow diagram (Figure 3). Patients were divided into two groups. Thirty-eight patients underwent the procedure using the eleven-bar cushion (eleven-bar, Wellness Korea, Chuncheon, Republic of Korea) for pelvic support (eleven-bar group), and 38 patients underwent the procedure with conventional pillow support (pillow group). Assignment to the eleven-bar group or pillow group was determined by the availability of the cushion in each procedure room, rather than by clinical characteristics or operator preference. Therefore, the allocation was non-random. Each eleven-bar cushion measures 10 cm in height, 10 cm in width, and 50 cm in length, and is composed of two separate pieces (Figure 4). In addition, it is composed of high-density polyurethane foam with a density of approximately 32–50 kg/m^3^ and Shore A 25–45 range of hardness. Place and use at appropriate intervals between two bars according to the patient’s pelvic size. We reviewed fluoroscopic images of the procedure and analyzed the VAS score, which was checked before and one month after the procedure.

### 2.1. Procedure

All procedures were performed by a pain-intervention specialist. The specialist was not aware that the procedures were part of a clinical study and performed all interventions as part of routine clinical practice. All procedures were performed without bed tilt, with patients breathing spontaneously, and without the use of sedation or muscle relaxants. The patients were placed in the prone position. While in the prone position, the eleven-bar group used an eleven-bar cushion positioned from the chest to the pelvis, whereas the pillow group used a standard pillow. After usual sterile preparation, the sacral hiatus was identified by palpation of the two sacral cornua and the interposed hiatal depression. Local anesthetic infiltration was carried out with a 25-gauge needle infiltrating 1% lidocaine about 2 mL. Twenty-gauge spinal needle (disposable nerve blockade needle, UNISIS corp., Tokyo, Japan) was inserted into sacral hiatus under fluoroscopic (OEC9800, General Electric, Boston, MA, USA) guidance and advanced to the cephalic direction. The needle was advanced and directed to cannulate the sacral canal. When needle placement was believed to be correct, aspiration was performed to exclude venous or dural puncture. Following this, 3 mL of contrast medium was injected once it was felt that the needle was in an appropriate position. If the needle was confirmed to be in the epidural space, an additional contrast medium, to bring the total to 10 mL, was injected into the epidural space. The injectate was administered manually at a steady rate without the use of any pressure-modulating devices. After that, 0.25% mepivacaine 8 mL and 2.5 mg dexamethasone was injected. Dexamethasone 2.5 mg was chosen as it falls within the typical 2–4 mg range commonly used for caudal epidural injections and is considered safe as a non-particulate steroid. Concomitant analgesic use was reviewed from medical records. Routine medications such as NSAIDs or acetaminophen were allowed and did not differ between the two groups.

### 2.2. Analysis of Fluoroscopic Images

**Figure 5:**
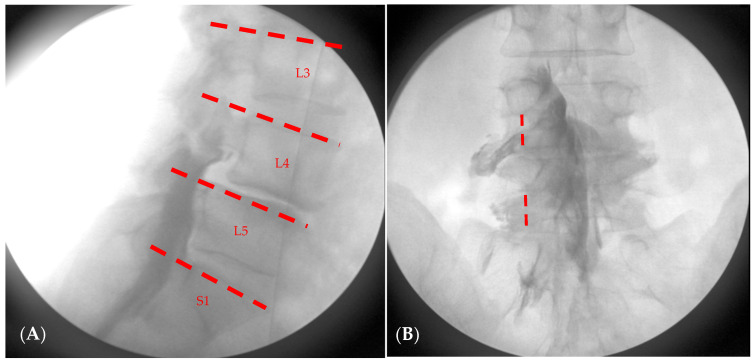
Schematic drawing for analysis of the level of epidural contrast spread in (**A**) and nerve root spread in (**B**). In (**A**), the dotted line indicates the range of epidural spread level. L3, includes the level above the upper margin of L4; L4 includes the level above the upper margin of L5; L5 includes the level between the upper margin of L5 and the L5-S1 intervertebral space; S1 includes the level between the upper margin of S1. In (**B**), the dotted line is in line with the center of pedicle. And if contrast crosses the line, it means nerve root spreading at that level. In this patient, the contrast medium reached L4 and spread to two nerve roots.

All radiographic findings were evaluated by an anesthesiologist from our pain clinic who was blinded to the group allocation and study hypothesis. The evaluator had access only to anonymized images without any clinical information, intervention details, or patient identifiers. The typical Christmas tree appearance in anteroposterior (AP) image without leaking into subcutaneous tissue, intrathecal space and vessel was considered as correct placement. The lateral image was obtained to identify the highest segment reached which is the primary end point of our study. The AP image was checked to identify the number of nerve roots spread (Figure 5).

### 2.3. Sample Size Determination and Statistical Analysis

By referring to the existing similar studies [8], with the significance level (α) = 0.05, power = 0.80, and dropout rate 10%, 38 eleven-bar groups and 38 pillow groups are required.

The highest segment contrast medium reaching, and the number of nerve root spread is compared by a Mann–Whitney U test between groups. For the highest level of contrast, numerical values were assigned as follows: L2 = 2, L3 = 3, L4 = 4, L5 = 5, and S1 = 6. The median and IQR were subsequently calculated based on these values. The VAS score changes after the caudal block, a secondary end point, is compared by a Mann–Whitney U test within and between groups. Additionally, regarding the correlation with the symptoms and history of surgery, the highest dye level was treated as a continuous variable and subjected to linear regression analysis, while the number of nerve root spreads was analyzed using Poisson regression based on frequency.

## 3. Results

Seventy-six patients underwent caudal block. Demographic data, including mean age, weight, height, symptoms, and history of spine surgery, are presented. There were no significant differences between the two groups in terms of patient demographics (Table 1).

**Figure 6:**
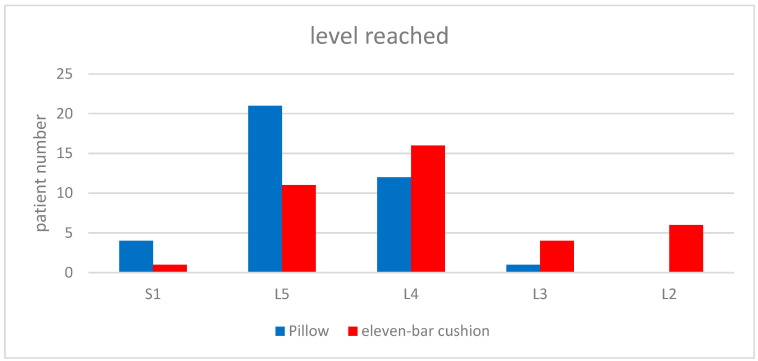
Spreading levels reached by contrast medium with eleven-bar cushion (red) and level reached with pillow (blue). There was a statistically significant difference.

On the Mann–Whitney U tests, the eleven-bar group demonstrated a significantly higher cephalic spread of contrast than the pillow group (the median highest dye level is four versus five; *p* < 0.001), while the number of nerve root opacification and VAS changes did not differ between groups (Table 2). For clinical interpretability, we additionally evaluated the proportion of patients whose contrast spread reached L3 or higher. This proportion was significantly greater in the eleven-bar group (10/38, 26.3%) than in the pillow group (1/38 and 2.6%; *p* = 0.0067, Fisher’s exact test) (Figure 6). In the ordinal logistic regression, the eleven-bar group showed a markedly increased likelihood of achieving higher cephalad levels (OR = 0.194), whereas prior lumbar surgery (OR = 4.245) and having both low back pain and radiating pain (OR = 0.063) were also significant predictors of dye spread pattern (Table 3). In contrast, Poisson regression demonstrated that age was the only variable associated with the number of nerve roots reached, with each 1-year increase associated with a 2% reduction in root opacification (IRR = 0.98; *p* = 0.003). Group, surgical history, and symptom category were not significant (Table 4).

## 4. Discussion

In this study, we investigated the use of an eleven-bar pelvic support cushion in the prone position on fluoroscopic epidural spread and evaluated the correlation between the level of spread, the number of roots involved, and analgesic efficacy in patients undergoing caudal block. Although the eleven-bar cushion increased the cephalad extent of epidural spread, this was not accompanied by better analgesic outcomes, with similar VAS reductions and nerve-root opacification between groups. Additional findings suggested that symptom pattern, surgical history, and age each contributed to differences in how the contrast spread within the epidural space.

No significant differences were observed in baseline demographic characteristics between the two groups. Beyond the positional effect of the eleven-bar cushion, patients’ symptom profiles also appeared to influence epidural distribution; those presenting with both low back pain and radiating pain tended to show a broader cephalic spread. In addition, advancing age was associated with a gradual reduction in the number of nerve roots opacified, suggesting an age-related limitation in epidural dispersibility.

Many studies investigated facilitating factors for contrast spreading level. Cleary et al. [8] investigated variation in Trendelenburg tilt on the cephalic distribution of the injectate. There was a trend for a 30° table tilt to extend the upper level reached by the caudal injection. Woo Seog Sim et al. [10] investigated the influence of needle gauge on fluoroscopic epidural spread and to assess the correlation between the spread level and analgesic efficacy in patients undergoing caudal block. Different needle gauges did not make significant differences of epidural spread and analgesic efficacy.

This study is meaningful in that it is the first study to confirm the change in the height of the contrast medium according to the eleven-bar cushion. To date, there have been no clinical studies directly investigating the relationship between abdominal pressure and contrast spreading. However, Visser, W Anton et al. [11] reported that the mid-thoracic epidural space pressure is higher than the low-thoracic epidural space pressure and suggested that this pressure gradient may influence the spread of contrast. Therefore, reducing abdominal pressure, thereby lowering the lumbar epidural space pressure, could increase the pressure gradient between the sacral and lumbar epidural spaces, potentially facilitating greater cephalic spread of contrast.

Spinal stenosis is more commonly observed in the lower lumbar spine, and the frequently pressed nerve roots are in the order of L5 (75%), L4 (15%), L3 (5.3%), and L2 (4%) [12]. Therefore, reaching L2 would generally cover the clinically relevant lumbar levels for most patients and is likely to be effective in most cases. This shows that the indication of caudal block, which was previously only used in low-level lesions, can be used at high levels when using an eleven-bar cushion.

This study had several limitations. First, as a retrospective analysis, important procedural variables—including injection volume, injection pressure, and anatomical variations—could not be fully controlled. Second, although cephalic spread differed between groups, we did not evaluate the quantitative relationship between the degree of contrast spread and VAS score changes. Third, factors known to affect epidural distribution, such as injectate viscosity, needle angle, patient BMI, and lumbosacral angulation, were not assessed in this study and may have contributed to variability in spread patterns. Fourth, the follow-up period after the caudal block was relatively short, and each patient was exposed to different analgesic regimens or interdisciplinary management strategies, which may have influenced post-procedure pain scores. Future prospective randomized studies with standardized injection protocols are required to address these limitations and validate our findings.

## 5. Conclusions

In this study, the use of an eleven-bar pelvic support cushion was associated with a higher cephalic level of epidural spread during caudal epidural injections. However, this increased spread did not result in significant differences in nerve root involvement or short-term analgesic outcomes. While the findings suggest a positional influence on contrast distribution, the clinical impact appears modest and remains to be clarified. Larger, well-designed prospective studies are needed to confirm the effect of pelvic support on epidural spread and to evaluate its potential benefits on analgesic outcomes and long-term efficacy.

## Figures and Tables

**Table 1:** Patient demographics.

	All Patients (n = 76)	Eleven-Bar Group (n = 38)	Pillow Group (n = 38)	*p*
Age (yr)	61.5 ± 16.5	60.4 ± 14.8	62.7 ± 18.1	0.553
Sex (M/F)	36/40	16/22	20/18	0.491
Height (cm)	162.5 ± 8.6	162.7 ± 8.6	162.3 ± 8.7	0.863
Weight (kg)	64.1 ± 12.4	64.1 ± 13.2	63.9 ± 11.6	0.927
Symptom				0.415
Low back pain	20 (26.3%)	9 (23.7%)	11 (28.9%)	
Radiating pain	37 (48.7%)	17 (44.7%)	20 (52.6%)	
Both	19 (25%)	12 (31.6%)	7 (18.4%)	
History of surgery				0.734
Yes	10 (13.2%)	4 (10.5%)	6 (15.8%)	
No	66 (86.8%)	34 (89.5%)	32 (84.2%)	

Values are expressed as numbers (%).

**Table 2:** Comparison of fluoroscopic outcomes and pain scores between groups (Mann–Whitney U test).

	All Patients (n = 76)	Eleven-Bar Group (n = 38)	Pillow Group (n = 38)	*p*
Fluoroscopic signals				
Highest dye level	4 (5−4)	4 (5−3.25)	5 (5−4)	<0.001 *
Number of nerve root spread	1 (1−0)	1 (1−0)	1 (1−0)	0.584
Pain severity (VAS)				
Before block	7 (8−6)	7 (8−6.25)	7 (8−6)	0.822
After block	5 (6−5)	5 (6−5)	5	0.691

Values are expressed as median (Q3–Q1); IQR = Q3–Q1. Numerical values for the highest dye level were assigned as follows: L2 = 2, L3 = 3, L4 = 4, L5 = 5, and S1 = 6. Values for the number of nerve root spread denote the count of opacified roots. The sign * denotes *p* < 0.05 by Mann–Whitney U test between the eleven-bar and pillow groups. VAS stands for visual analogue scale.

**Table 3:** Multivariable ordinal logistic regression analysis for factors associated with highest dye level.

Variable	Multivariable	
	OR (95% CI)	*p*
Age	1.017 (0.983–1.053)	0.327
Sex		
Female	-	-
Male	1.227 (0.341–4.529)	0.755
Height	1.006 (0.924–1.094)	0.895
Weight	1.018 (0.977–1.061)	0.406
Group		
Pillow	-	-
11 bar cushion	0.194 (0.071–0.499)	<0.001 *
History of surgical		
No	-	-
Yes	4.245 (1.056–18.681)	0.046
Symptom		
No pain	-	-
Low back pain	0.518 (0.121–2.168)	0.368
Radiating pain	0.324 (0.071–1.426)	0.137
Both	0.063 (0.012–0.305)	<0.001 *

Values are presented as odds ratios (ORs) with 95% confidence intervals (CIs), obtained from an ordinal logistic regression model. The reference categories were female for sex, pillow group for treatment group, no history of surgery for surgical history, and no pain for symptom type. The sign * denotes *p* < 0.05.

**Table 4:** Univariable and multivariable Poisson regression analysis for factors associated with the number of nerve roots reached.

Variable	Univariable		Multivariable	
	IRR (95% CI)	*p*-Value	IRR (95% CI)	*p*-Value
Age	0.98 (0.96 to 0.99)	0.003 *	0.98 (0.96–0.99)	0.003 *
Gender				
Female	-	-		
Male	0.63 (0.36 to 1.09)	0.105		
Height	1.00 (0.97 to 1.03)	0.934		
Weight	1.00 (0.97 to 1.02)	0.654		
group				
Pillow	-	-	-	-
11 bar cushion	1.20 (0.71 to 2.06)	0.501	1.10 (0.64–1.90)	0.732
History of surgery				
No	-	-	-	-
Yes	0.66 (0.23 to 1.50)	0.376	0.82 (0.28–1.92)	0.686
Symptom				
Low back pain	-	-	-	-
Radiating pain	0.97 (0.51 to 1.91)	0.916	1.02 (0.53–2.02)	0.957
Both	1.20 (0.59 to 2.50)	0.614	1.47 (0.69–3.18)	0.315

Values are presented as incidence rate ratios (IRRs) with 95% confidence intervals (CIs). Multivariable Poisson regression included variables with clinical relevance or *p* < 0.10 in univariable analysis. The reference categories were female (sex), pillow group, no history of surgery, and low back pain (symptom type). The sign * denotes *p* < 0.05.

## Data Availability

The data presented in this study are available upon request from the corresponding author due to privacy and ethical restrictions. The data are not publicly available.

## Abbreviations

The following abbreviations are used in this manuscript:

CESI	Caudal Epidural Steroid Injection
VAS	Visual Analogue Scale
AP	Anteroposterior (fluoroscopic view)
LBP	Low Back Pain
